# Community attitudes on tuberculosis in Botswana: an opportunity for improving the National Tuberculosis Programme outcomes, 2011

**DOI:** 10.1186/s13104-018-3585-1

**Published:** 2018-07-23

**Authors:** Godfrey Musuka, Vonai Teveredzi, Lesego Busang, Innocent Chingombe, Panganai Makadzange, Setshwano Mokgweetsinyana, Ronald Ncube, Julita Maradzika, Carmillo Fungai Chinamasa, Themba Moeti

**Affiliations:** 1 African Comprehensive HIV/AIDS Partnerships, Gaborone, Botswana; 2 ICAP at Columbia University, Harare, Zimbabwe; 3 Ark Foundation, Harare, Zimbabwe; 4 Ministry of Health, Gaborone, Botswana; 5 Department of Community Medicine, University of Zimbabwe, Harare, Zimbabwe; 6 Health Systems Trust, Durban, South Africa

**Keywords:** Tuberculosis, Botswana, HIV, Community, Knowledge, Attitudes

## Abstract

**Objectives:**

The Botswana tuberculosis HIV Knowledge Attitude and Practice study sought to assess knowledge, attitudes and practices of communities on TB and identify sources of their information on this disease and HIV. Specific objectives of the study were to: (a) collect baseline information on the knowledge, attitudes, and practices about tuberculosis treatment seeking and adherence behaviors in Botswana. (b) Identify barriers which discourage people who may have smear positive tuberculosis from testing and getting treatment (e.g. social stigma) and constraints which prevent them from initiating and completing treatment.

**Results:**

Approximately 92% of respondents (n = 2029), reported that having TB was not something embarrassing, while about 97% (n = 2030) were not ashamed of having a family member with TB. Approximately 95% (n = 2030) expressed willingness to accommodate their relatives with TB at their homes or, work with TB patients (n = 2026). About 21% of the respondents however, believed in myths that TB infection is a result of either having sex with women who had miscarried (n = 2028), or food poisoning (n = 2031) while about 17% believed that TB infection is a result of sleeping with a widow or widower (n = 2031).

## Introduction

Botswana, like many other African countries has not been spared from the burden of tuberculosis [1]. Though declining, TB case notification rates remain high in Botswana and rank sixth globally [1]. The TB case notification rates for the country reached a peak of > 600 per 100,000 population in 2002 before starting to decline slightly to an average of > 400 per 100,000 from 2007 to 2009 (MOH Botswana). WHO estimated a tuberculosis incidence rate of 414 per 100,000 population and a tuberculosis prevalence rate (all forms) of 326 per 100,000, in 2017.^1^ The high number of TB cases creates a strain on the health facilities and also poses a risk of the spread of tuberculosis infection in families and communities. These high case notification rates are mainly attributed to high HIV prevalence in the country, where 60–80% of tuberculosis patients are co-infected with HIV [2].

In order to strengthen the TB/HIV control in the country, the Botswana Ministry of Health (MoH) undertook to review the National TB Programme Strategic and Implementation Plans for the period 2012–2016. The overall goal of the revised plans is to reduce the morbidity, mortality and psycho-socio-economic burden and impact associated with the disease. To ensure that this process is based on evidence, the Botswana National TB Programme (BNTP), with support from the African Comprehensive HIV/AIDS Partnerships (ACHAP) conducted a TB/HIV (Knowledge Attitude and Practice) KAP study in 2011 to assess knowledge, attitudes and practices of communities on TB and identify sources of their information on this disease and HIV.

## Main text

### Study design

This is a descriptive cross sectional quantitative and qualitative study, based on methodological triangulation (the combination of various methods and data sources to refine and test particular interpretations and assumptions of TB at community level and in the health care system. This study combined both quantitative qualitative participatory approaches to collecting information related to TB among various groups.

### Study sites

The study was conducted in the four main geographic regions of Botswana—the south-east, central/north-east, north-west (Ngamiland, Chobe), and western (Gantsi, Kgalagadi) to capture a good mix of ethnic groupings. Two health districts from each of these four regions were selected. The aim was to provide a range of study sites distributed across the country. The 8 health districts represent roughly 28.5% of the 28 health districts in Botswana’s health system. The 8 districts were considered as manageable within the resource and time constraints of the study.

Site selection also factored in a mix of different sizes of rural and urban settlements (i.e. cities, major villages and smaller settlements). Selection of the 8 health districts was based on TB prevalence rates, geographical location and population density resulting in two cities (Gaborone and Francistown), two major villages/townships (Gantsi, Maun), and four communities within the 500–5000 population range (i.e. Boteti, Kweneng East, Kgalagadi North and Tutume), located in four other health districts distributed across the country were also targeted for the study.

### Data collection

Data collecting from households members was collected using an interviewer-administered questionnaire. Once selected, and a respondent consented to be interviewed, the interview was conducted in private by trained researchers who were supervised by TB/HIV Officers from the Botswana Ministry of Health. Interviews were done in either English or Setswana and recorded on the interview questionnaires by the Interviewer. Information collected through the household interviews; include demographics, attitudes, knowledge including myths and misconceptions on TB.

### Respondent eligibility criteria

To participate in the study, respondents needed to satisfy the following eligibility criteria;

- Be adult aged 18 years or above.
- Able to speak English or Setswana.
- Able and willing to consent on the day of the interview.

### Sampling and sample size determination for the Household Survey

The selection of the study areas and sampling framework involved decision-making at four levels:

- Purposive selection of 8 health districts to be studied in the four main regions of Botswana.
- Selection of 14 location(s) to be studied within each health district.
- Selection of households to be visited within each location, and;
- Selection of the 2000 persons to be invited for interview in each household visited.

A predetermined sample size of 2000 respondents was regarded as adequate within the time and resource available to give a representative data for analysis in this survey. An estimate of 45% of potential refusal and empty households was used to calculate the desired sample size. This number would ensure that an adequate number of persons would be drawn from the sampling frame to account for an estimated amount of non-response (refusal to participate, empty houses, etc.). An estimate of 45% of potential refusal and empty households was used to ensure that the desired sample size 2000 was reached at the end of the survey. In addition, this number was considered manageable within the limited time and budget.

### Data management, processing and analyses

Data from the Botswana TB KAP Household survey were transferred from paper copies of the household questionnaires and entered on computer using the SPSS version 19 software (IBM, 2011) by 4 Data Entry clerks formally employed by ACHAP for that purpose and supervised by an Monitoring and Evaluation Specialist with Statistical and Data Management training and experience. After entry all data were checked and cleaned by the data entry team in preparation for analysis.

### Findings

Out of the 2032 participants 67% were females while the remaining 33% were males. The age range for the participants was from 18 to 94 years, and the mean age was 34.8 years while the median age was 31 years. The main languages spoken by participants at home, were Setswana (65.8%), Kalanga (16.9%), Sekgalagadi (11.8%), English (2.6%) and other languages combined, comprising of Sesarwa, Shona, Seherero, Ndebele, Sebirwa and Sembukushu (2.9%).; About 60% of the interviews took place in the rural areas while the remainder, in the urban areas. The majority of the respondents had completed junior secondary (32%), followed by senior secondary (26%).

### Attitudes towards TB patients

The figure below shows respondents self-reported acceptance of different TB scenarios. It clearly shows low levels of TB stigmatization. Unfortunately, the indicator on myths about TB shows the existence of some substantially high levels of misconceptions on TB, within communities in Botswana. Disaggregation of these results is discussed in the analysis below (Table 1).

**Table 1:** Below shows the frequency of respondents in agreement with some attitudes towards TB patients

Attitude measure	Denominator	% agreeing
Willingness to work with someone who has TB	2026	95
Not ashamed if a family member is TB positive	2030	97
Willing to accommodate a family member with TB	2030	95
It’s not embarrassing for one to have TB	2029	92

### It’s embarrassing to have TB

Approximately 92% of respondents (n = 2029), felt that having TB is not something that one should be embarrassed of. Within sex categories, female respondents (n = 1350) were more likely, than male respondents (n = 679), to report that it is not embarrassing for one to be diagnosed of TB, as 93% compared 90% reported the same, respectively. The same response was slightly higher among rural respondents (n = 1225) compared to urban residents (n = 804) with 93% and 90%, respectively.

Similar views were echoed by respondents across the respondents’ highest level of education attained, with 94% among those respondents with secondary education (n = 1180), followed by respondents with tertiary education (n = 325) as well as those respondents with no level of education attained (n = 168), each with 91%. Respondents with primary level education (n = 356), were lowest with only 86%, reporting the same.

### It’s shameful to have a family member with TB

About 97% of respondents (n = 2030), reported that it is not shameful to have a family member with TB. Both females (n = 1351) and female (n = 679) were in concurrence with the fact that having a TB family member with TB is not something they would feel ashamed of, with 98 and 97% reporting the same, respectively. There was also a high concurrence to this view across the rural (n = 1226) and urban (n = 804) respondents, with respondents from the rural areas however, having a slightly higher concurrence rate (98%), than their urban counterparts (97%). Across the different levels of education attained, concurrence with this view was highest among the respondents with tertiary level of education (n = 325), with approximately 100%, followed by respondents with secondary education (n = 1180) with 98% and lastly, respondents with primary education (n = 356) as well as those with no schooling (n = 169), each with approximately 95%.

In addition, respondents from the various religious backgrounds who participated in the study were generally in agreement with the view that having a family member with TB is not something one should feel ashamed of. Christians (n = 1622) had the highest concurrence rate to this view (98%), followed by those respondents with no religion (n = 317) with 96%, and lastly respondents belonging to the other categories of religions combined (n = 90) with 93%.

### Willingness to accommodate a TB positive family member in the house

Approximately 95% of the respondents (n = 2030) reported that they would accommodate their TB positive relatives in their homes, with females (n = 1351) being slightly higher with 96%, than males (n = 679) with 95%, reporting the same. Respondents from the rural areas (n = 1226) were slightly more willing to accommodate a family member with TB in their home than their urban counterparts with 96 and 95%, respectively, reporting the same.

More than 90% of the respondents across all the educational levels were in agreement that they would accommodate a family member who has TB, in their homes. Respondents who had completed tertiary level of education (n = 325) had the highest concurrence with 99%, followed by those with secondary level education (1180) with 96%, then respondents with no schooling (n = 169) with 92% and lastly, respondents that have attained primary level of education (n = 356), with 90%.

This same willingness was above 90% across the respondents from the different religious groups that took part in the study. It was, highest among the Christians (n = 1622) with 96%, followed by those respondents with no religion (n = 317) with 93% and lastly those on the other category of religions (n = 90) with 91%.

### Willingness to work with a TB positive patient

Apart from accommodating a family member with TB in their homes, 95% (n = 2026) reported that they would accept working with someone with TB. Females (96%) were slightly more agreeable to this view, than men (95%). Rural/Urban comparison shows that 96% of respondents from the rural areas (n = 1224) compared to 95% from the urban areas (n = 804), were willing to work with a TB patient.

Approximately 99% of respondents with tertiary level education (n = 325) were accommodative of having a workmate with TB, followed by those with secondary level education (n = 1177) with 97%. Ninety-one percent and 87% of respondents with no schooling (n = 169) as well as those who attained primary level education (n = 355), respectively, also reported that they would accept having a TB positive workmate.

### Myths/misconceptions

About 21% of the respondents, believed that TB infection is a result of either having sex with women who had miscarried (n = 2028), or food poisoning (n = 2031) while about 17% believed that it is a result of sleeping with a widow or widower (n = 2031) (Table 2).

**Table 2:** Frequency of respondents in agreement a statement on myths about contracting TB

Myth	Denominator	% agreeing
TB is a result of Poisoning (Ke sejeso)	2031	21
TB is a result of sleeping with a widow or widower	2031	17
TB is a result of having sex with a woman who has had a miscarriage	2028	21

Our study findings show that communities in Botswana, both rural and urban are highly accommodative of TB patients. This high acceptance of people with TB has been demonstrated across the various socio-demographic characteristics of the respondents such as age, sex, highest levels of education attained and religious affiliation. However, our study also showed the existence of myths which may negatively affect the implementation and success of the TB programme at community level. There is need to invest in health education to improve the understanding of individuals and communities. Based on the above findings, it is critical for the Botswana National Tuberculosis Programme to promote and support the positive attitudes found in communities in order to enhance treatment outcomes.

The national programme has an opportunity to increase the number of TB patients on community organized and run initiatives for TB patients and relieve the pressure on health care facilities.

## Limitations

Limitations of our study include the fact that, participants might have had recall bias as data was collected about their past experiences. In addition, the study could not allow cause-effect relationship analysis because of its cross-sectional nature. There are chances of people reporting what they do not practice.

## Abbreviations

ACHAP: African Comprehensive HIV/AIDS Partnerships
TB: tuberculosis
HIV: human immunosuppression virus
DOT: direct observational treatment
KAP: Knowledge Attitude and Practice
MOH: Ministry of Health Botswana
BNTP: Botswana National Tuberculosis Programme

## Footnotes

1 WHO 2017 Global TB Report.
